# N-terminal Pro-B-Type Natriuretic Peptide (NT-proBNP) Versus Artificial Intelligence-Enabled Electrocardiography for the Diagnosis of Acute Heart Failure in Emergency Departments: A Systematic Review

**DOI:** 10.7759/cureus.115927

**Published:** 2026-09-08

**Authors:** Lamyaa A Elhassan, Mohamed Abdelrahman Mohamed Ali, Elamein Yousif, Mahmoud Abdelghany Eldyasty Ahmed Eid, Hiba Elsadig Abdelmonim Ahmed, Nada Fathi F Mohanmed Ahmed Hamid, Asmaa Fadlallah Hammad Atroun, Mohamed Mahmoud Ismaeel Mohamed, Hanady ME M Osman

**Affiliations:** 1 Basic Medical Sciences, Jazan University, Jazan, SAU; 2 Internal Medicine, Portiuncula University Hospital, Ballinasloe, IRL; 3 Internal Medicine, Al-Ahli Hospital, Doha, QAT; 4 Cardiology, Royal Cornwall Hospitals National Health Service (NHS) Trust, Truro, GBR; 5 Acute Internal Medicine, Peterborough City Hospital, Peterborough, GBR; 6 Dermatology, Al-Azhar University, Cairo, EGY; 7 General Practice, National Ribat University, Khartoum, SDN; 8 Internal Medicine, Sulaiman Al Habib Medical Group, Riyadh, SAU; 9 Internal Medicine, Health Oasis Hospital, Riyadh, SAU; 10 General Practice, University of Bahri, Khartoum, SDN; 11 Quality Improvement and Patient Safety, Najran Armed Forces Hospital, Ministry of Defense Health Services, Najran, SAU

**Keywords:** acute heart failure, artificial intelligence, deep learning, diagnostic accuracy, electrocardiography, emergency department, nt-probnp, systematic review

## Abstract

N-terminal pro-B-type natriuretic peptide (NT-proBNP) is the guideline-endorsed biomarker for diagnosing acute heart failure (AHF) in the emergency department (ED), while artificial intelligence-enabled electrocardiography (AI-ECG) has emerged as a rapid, phlebotomy-free alternative. AI-ECG models are trained end-to-end on raw waveform or ECG image data and learn subtle depolarisation, repolarisation and conduction features that are not recognisable by conventional criteria; most were trained to detect left ventricular systolic dysfunction (LVSD) rather than the AHF syndrome itself. Their relative performance has not been systematically appraised.

Following Preferred Reporting Items for Systematic Reviews and Meta-Analyses (PRISMA) 2020, four databases were searched from January 2020 to August 2026 for original primary studies reporting the diagnostic accuracy of NT-proBNP and/or AI-ECG for AHF, or its structural correlate LVSD, in adult ED patients. Full database-specific search strategies are reported. Risk of bias was assessed with Quality Assessment of Diagnostic Accuracy Studies 2 (QUADAS-2) and reporting completeness of the artificial intelligence studies with the Checklist for Artificial Intelligence in Medical Imaging (CLAIM) 2024. Heterogeneity in target conditions, thresholds and reference standards precluded meta-analysis, and comparisons were classified a priori as direct (within-cohort) or indirect (between-study).

Eleven studies comprising more than 40,000 ED encounters were included. NT-proBNP discriminated adjudicated AHF consistently across large prospective cohorts (area under the curve (AUC) 0.87-0.92), with excellent rule-out performance at 300 pg/mL (sensitivity 96%-99%, negative predictive value 95%-98%) but modest specificity (54%-56%). AI-ECG achieved AUCs of 0.88-0.94, highest for LVSD rather than adjudicated AHF, and exceeded NT-proBNP in all four direct within-cohort comparisons (0.89 vs 0.80; 0.91-0.92 vs 0.75). In contrast to the standardised NT-proBNP decision limits, only three of seven AI-ECG studies reported an explicit operating threshold, and the models were trained on four different target labels. Eight studies were at low and three at high risk of bias. CLAIM appraisal showed complete reporting of study design and evaluation but incomplete reporting of model specification, data partitioning and data availability.

NT-proBNP remains the better-validated test for adjudicated AHF and retains unmatched rule-out value, whereas AI-ECG offers faster and cheaper detection of structural substrate. Because no study compared the two tests against an adjudicated AHF reference standard, they appear complementary rather than interchangeable, and prospective head-to-head and outcome-based studies are required.

## Introduction and background

Acute heart failure (AHF) is one of the commonest causes of emergency hospitalisation in older adults and accounts for a large share of unscheduled cardiovascular care worldwide [1,2]. The clinical challenge in the emergency department (ED) is not the management of established heart failure but the rapid separation of AHF from the many alternative causes of acute dyspnoea. History, examination, chest radiography and the unaided electrocardiogram (ECG) each lack adequate sensitivity and specificity in isolation, and diagnostic uncertainty at the point of disposition is independently associated with longer hospitalisation and worse outcomes [3]. A test that resolves this uncertainty within the first minutes of the ED encounter therefore carries considerable clinical value.

Natriuretic peptide measurement has been the reference solution for two decades. The The N-terminal Pro-BNP Investigation of Dyspnea in the Emergency department (PRIDE) study established age-stratified N-terminal pro-B-type natriuretic peptide (NT-proBNP) decision limits in undifferentiated dyspnoeic patients [4], and the International Collaborative of N‑terminal pro‑B‑type Natriuretic Peptide Re‑Evaluation of Diagnostic Cut‑Offs in the Emergency Department (ICON-RELOADED) study confirmed that an age-independent threshold of 300 pg/mL reliably excludes AHF while thresholds of 450, 900 and 1,800 pg/mL support rule-in [5]. These values are embedded in contemporary guidelines [1]. NT-proBNP nonetheless has recognised limitations: specificity is degraded by advanced age, atrial fibrillation and renal impairment, sensitivity is attenuated by obesity and by preserved ejection fraction, a substantial proportion of results fall within an intermediate grey zone, and the test requires venepuncture, a laboratory analyser and a turnaround time that frequently exceeds an hour.

Artificial intelligence-enabled electrocardiography (AI-ECG) offers a conceptually different answer to the same question. Unlike conventional electrocardiographic criteria, which depend on a small number of human-defined measurements such as QRS duration, voltage or axis, AI-ECG models are trained end-to-end on the digitised waveform or on an image of the printed tracing. Convolutional, residual and transformer architectures learn distributed patterns across all 12 leads - subtle alterations of QRS morphology and fragmentation, repolarisation and T-wave abnormality, conduction delay and beat-to-beat variability - that reflect chamber dilatation, hypertrophy and myocardial fibrosis but that are individually too small or too diffuse for a human reader to detect. The seminal Mayo Clinic model, trained on more than 44,000 patients, identified an ejection fraction ≤35% from the resting 12-lead ECG with an area under the curve of 0.93 [6], and comparable performance has since been reproduced by independent groups. The model output is a continuous probability or a predicted ejection fraction, dichotomised at a threshold that the developer, rather than a guideline committee, selects.

This distinction matters for the emergency question at hand. What most AI-ECG algorithms detect is left ventricular systolic dysfunction (LVSD), a chronic structural abnormality, whereas AHF is an acute clinical syndrome of congestion that may occur with a normal ejection fraction and that is frequently absent in patients who have stable chronic LVSD. LVSD is therefore neither necessary nor sufficient for AHF, and an algorithm that detects it accurately may still misclassify the breathless patient. Whether AI-ECG performance translates into accuracy for AHF specifically remains contested, and no previous review has directly juxtaposed the two index tests in the emergency setting in which they compete. We therefore systematically reviewed primary studies evaluating NT-proBNP and AI-ECG for the diagnosis of AHF in adults presenting to EDs, in order to quantify the performance of each test, to establish what each AI-ECG model was actually trained to detect and at what threshold, to appraise the available head-to-head comparisons, and to define the methodological gaps that must be closed before AI-ECG can be positioned as an alternative to natriuretic peptide testing.

## Review

Materials and methods

Study Design and Protocol

This review was designed, conducted and reported in accordance with the Preferred Reporting Items for Systematic Reviews and Meta-Analyses (PRISMA) 2020 statement [7]. A written protocol specifying the review question, eligibility criteria, search strategy, data items, quality-appraisal instruments and synthesis approach was agreed by the review team before searching commenced and was followed without amendment. The protocol was not registered in an international prospective register such as PROSPERO; this is stated explicitly here and acknowledged among the limitations, and the protocol is available from the corresponding author on request. No ethical approval was required because only previously published aggregated data were used.

Eligibility Criteria

Eligibility was specified a priori using the Population, Intervention, Comparison, Outcomes and Study (PICOS) framework, adapted for diagnostic accuracy studies by substituting index test and reference standard for intervention and outcome, in line with established guidance for reviews of diagnostic test accuracy [8]; the comparator element was retained because the review question is explicitly comparative. We included original peer-reviewed primary studies of adults (≥18 years) presenting acutely to an ED that evaluated NT-proBNP and/or an AI-ECG algorithm against an explicit reference standard for AHF or for LVSD and reported at least one quantitative accuracy measure, and we excluded non-original publication types, non-emergency settings, purely prognostic evaluations and reports published before 2020. Full criteria are given in Table 1. The date restriction is substantively justified: the first ED evaluation of AI-ECG appeared in 2020, so the comparative literature is in practice confined to the last five to six years, and contemporaneous NT-proBNP studies ensure that both index tests are judged over the same era of assay technology, case mix and guideline thresholds.

**Table 1:** Eligibility criteria (PICOS framework adapted for diagnostic test accuracy) AHF: acute heart failure; AI-ECG: artificial intelligence-enabled electrocardiography; AUC: area under the receiver operating characteristic curve; BNP: B-type natriuretic peptide; ECG: electrocardiogram; ED: emergency department; ICU: intensive care unit; LVSD: left ventricular systolic dysfunction; MR-proANP: mid-regional pro-atrial natriuretic peptide; NPV: negative predictive value; NT-proBNP: N-terminal pro-B-type natriuretic peptide; PICOS: Population, Intervention, Comparator, Outcomes, Study design; PPV: positive predictive value.

Domain	Included	Excluded
Population	Adults ≥18 years presenting acutely to an ED with dyspnoea, suspected AHF or an undifferentiated presentation for which a 12-lead ECG was obtained.	Children; pregnant women; outpatients; ward, ICU or screening cohorts; established AHF at enrolment.
Index test	NT-proBNP immunoassay on presentation and/or an AI-ECG algorithm applied to 12-lead ECG waveform or image data.	BNP or MR-proANP alone; rule-based ECG criteria; AI applied only to non-ECG modalities.
Comparator	The alternative index test, an alternative assay or threshold, another diagnostic modality, or none (stand-alone accuracy).	No extractable accuracy estimate for an eligible index test.
Reference standard and outcomes	Adjudicated or documented AHF diagnosis, or echocardiographic LVSD; at least one accuracy measure (AUC, sensitivity, specificity, PPV, NPV or likelihood ratio).	Prognostic outcomes only; biomarker concentrations or model scores reported without accuracy metrics.
Study design	Original peer-reviewed primary diagnostic accuracy, cohort or cross-sectional studies, in English, published January 1, 2020 to August 21, 2026.	Reviews, meta-analyses, conference abstracts, editorials, letters, case reports, protocols, preprints; duplicate reports of one cohort.

Search Strategy

PubMed, Scopus, Web of Science and Embase were searched from January 1, 2020 to August 21, 2026, supplemented by citation searching of the reference lists of all included articles and of relevant recent reviews. The search combined three concept blocks with the Boolean operator AND: the target condition, the index tests, and the emergency setting. Controlled vocabulary (Medical Subject Headings in PubMed, Emtree terms in Embase) was combined with free-text terms, and truncation was used to capture variant word endings. Language and date limits were applied within each interface. The complete database-specific search strings, exactly as executed, are reproduced in Table 2 so that the search can be replicated or updated.

**Table 2:** Database-specific search strategies as executed Searches were executed on August 21, 2026. Concept blocks were combined with the Boolean operator AND and terms within blocks with OR; truncation (*) captured variant word endings. The record counts correspond to those shown in Figure 1. ECG: Electrocardiogram; EKG: electrocardiogram; Mesh: Medical Subject Headings; NT-proBNP: N-terminal pro-B-type natriuretic peptide; TS: topic search; ti,ab,kw: title, abstract and keyword fields.

Database (interface)	Search string	Limits	Records
PubMed (NCBI)	("Heart Failure"[Mesh] OR "heart failure"[tiab] OR "acute heart failure"[tiab] OR "cardiac failure"[tiab] OR "Dyspnea"[Mesh] OR dyspnoea[tiab] OR dyspnea[tiab] OR "Ventricular Dysfunction, Left"[Mesh] OR "left ventricular systolic dysfunction"[tiab]) AND ("Natriuretic Peptide, Brain"[Mesh] OR "NT-proBNP"[tiab] OR "N-terminal pro-B-type natriuretic peptide"[tiab] OR "natriuretic peptide"[tiab] OR "Artificial Intelligence"[Mesh] OR "Deep Learning"[Mesh] OR "Machine Learning"[Mesh] OR "artificial intelligence"[tiab] OR "deep learning"[tiab] OR "machine learning"[tiab] OR "neural network*"[tiab] OR "Electrocardiography"[Mesh] OR electrocardiograph*[tiab] OR ECG[tiab] OR EKG[tiab]) AND ("Emergency Service, Hospital"[Mesh] OR "emergency department*"[tiab] OR "emergency room*"[tiab] OR "emergency service*"[tiab] OR triage[tiab] OR "acute care"[tiab])	2020/01/01 to 2026/08/21; English	93
Scopus (Elsevier)	TITLE-ABS-KEY(("heart failure" OR "acute heart failure" OR "cardiac failure" OR dyspnoea OR dyspnea OR "left ventricular systolic dysfunction") AND ("NT-proBNP" OR "natriuretic peptide" OR "artificial intelligence" OR "deep learning" OR "machine learning" OR "neural network*" OR electrocardiograph* OR ECG OR EKG) AND ("emergency department" OR "emergency room" OR "emergency service" OR triage OR "acute care")) AND PUBYEAR > 2019 AND (LIMIT-TO(LANGUAGE, "English"))	2020 to 2026; English	86
Web of Science Core Collection (Clarivate)	TS=(("heart failure" OR "acute heart failure" OR "cardiac failure" OR dyspnoea OR dyspnea OR "left ventricular systolic dysfunction") AND ("NT-proBNP" OR "natriuretic peptide" OR "artificial intelligence" OR "deep learning" OR "machine learning" OR "neural network*" OR electrocardiograph* OR ECG OR EKG) AND ("emergency department" OR "emergency room" OR "emergency service" OR triage OR "acute care"))	Timespan 2020 to 2026; English	82
Embase (Elsevier)	('heart failure'/exp OR 'heart failure':ti,ab,kw OR 'acute heart failure':ti,ab,kw OR 'dyspnea'/exp OR dyspnoea:ti,ab,kw OR 'left ventricular systolic dysfunction':ti,ab,kw) AND ('brain natriuretic peptide'/exp OR 'nt probnp':ti,ab,kw OR 'natriuretic peptide':ti,ab,kw OR 'artificial intelligence'/exp OR 'deep learning'/exp OR 'machine learning'/exp OR 'neural network*':ti,ab,kw OR 'electrocardiography'/exp OR electrocardiograph*:ti,ab,kw OR ecg:ti,ab,kw) AND ('emergency ward'/exp OR 'emergency department':ti,ab,kw OR 'emergency room':ti,ab,kw OR triage:ti,ab,kw OR 'acute care':ti,ab,kw)	(2020-2026)/py; [english]/lim	65
Citation searching	Backward and forward citation tracking of the reference lists of all included articles and of relevant recent reviews.	2020 to 2026; English	12
Total	—	—	338

Study Selection

All records retrieved from the four databases were exported to a reference manager and de-duplicated electronically, with residual duplicates removed by hand. Two reviewers then independently screened every title and abstract against the eligibility criteria in Table 1, using a liberal-accelerated approach in which a record was retained for full-text review if either reviewer judged it potentially eligible. The full texts of all retained records were retrieved and independently assessed by the same two reviewers, each of whom recorded a primary reason for exclusion from a prespecified hierarchy (not an emergency population; not an eligible index test; target condition not AHF or LVSD; ineligible publication type; no extractable accuracy estimate; duplicate cohort). Disagreements at either stage were resolved by discussion, and any that persisted were adjudicated by a third reviewer. Records identified by citation searching were screened through the same two-stage process. The flow of records, the number excluded at each stage and the reasons for exclusion at the full-text stage are shown in the PRISMA 2020 flow diagram (Figure 1).

Data Extraction

Two reviewers independently extracted, using a pilot-tested form, the first author, year, country, number of centres, design, enrolment period, eligibility criteria, sample size and prevalence of the target condition, the index test with its assay platform or algorithm architecture, the model input (waveform or image) and the decision threshold applied, the comparator, the reference standard and its method of ascertainment, the interval between index test and reference standard, and all reported accuracy measures with confidence intervals. Because the review question turns on what each algorithm was trained to detect, the target label used during model development and the operating threshold used at validation were extracted as separate, explicitly recorded fields. Discrepancies were resolved against the source article.

Quality Appraisal

Methodological quality and applicability were appraised with the Quality Assessment of Diagnostic Accuracy Studies 2 (QUADAS-2) tool across its four risk-of-bias domains [9]. Because seven included studies evaluated a machine-learning index test, the reporting completeness of these studies was additionally appraised against the Checklist for Artificial Intelligence in Medical Imaging (CLAIM) 2024 update, the current consensus reporting standard for medical artificial intelligence research [10]. CLAIM items were grouped into eight domains, and each domain was judged as fully reported, partially reported, not reported or not applicable. Both appraisals were performed independently by two reviewers, with disagreements resolved by consensus.

Data Synthesis

A structured narrative synthesis was undertaken, with results tabulated separately for NT-proBNP, for AI-ECG and for the studies providing a within-cohort head-to-head comparison. Meta-analysis was not performed. This decision was made a priori and reflects heterogeneity across three dimensions that statistical pooling cannot reconcile: the target condition differed between studies (adjudicated AHF, cardiac versus non-cardiac dyspnoea, and echocardiographic LVSD at ejection fraction thresholds of ≤35% or ≤40%); the index tests comprised distinct NT-proBNP immunoassays and mutually non-exchangeable neural network architectures; and decision thresholds were variously prespecified, guideline-derived or data-derived. Pooling accuracy estimates across studies whose reference standards define different diseases would have produced a precise but uninterpretable summary statistic.

To keep the strength of inference explicit, every comparison reported in this review is classified as either direct or indirect. A direct comparison is one in which both index tests were applied to the same patients, verified against the same reference standard, and compared with a paired statistical test; only these support inference about relative accuracy. An indirect comparison is one in which accuracy estimates from separate cohorts are placed side by side; because such estimates differ in disease prevalence, spectrum, reference standard and threshold, they are descriptive only and are not treated as evidence of superiority. Reported p-values for differences between areas under the curve are presented as published, and no new statistical testing was undertaken.

Results

Study Selection

Database searching identified 326 records (PubMed 93, Scopus 86, Web of Science 82, Embase 65) and citation searching identified a further 12. After the removal of 187 duplicates, 139 records were screened for relevance and 79 were excluded. Of the 60 database-derived reports sought for retrieval, six could not be obtained and 54 were assessed in full text; 45 were excluded because they were not based on acute heart failure (n=14), were reviews, editorials or commentaries (n=19), or were preprints or opinion pieces (n=12), leaving nine eligible reports. Of the 12 reports identified by citation searching, one could not be retrieved and 11 were assessed in full text, of which nine were excluded for an ineligible study design (n=7) or an ineligible index test (n=2), leaving two eligible reports. Eleven studies were therefore included in the qualitative synthesis (Figure 1).

**Figure 1:**
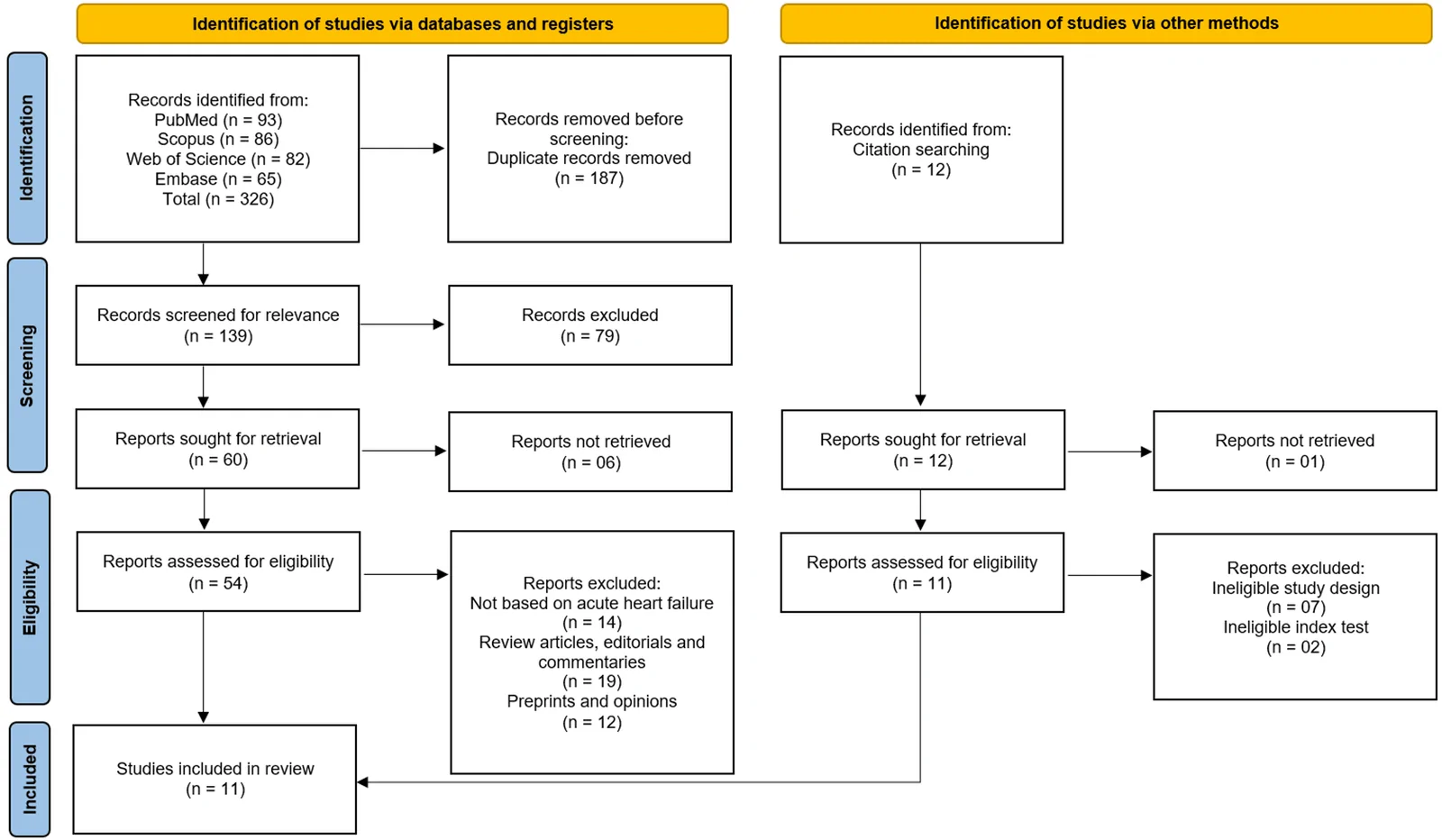
PRISMA 2020 flow diagram of study identification, screening, eligibility assessment and inclusion PRISMA: Preferred Reporting Items for Systematic Reviews and Meta-Analyses

Characteristics of the Included Studies

The characteristics of the 11 included studies are summarised in Table 3. In chronological order these were a Mayo Clinic emergency department (ED) dyspnoea cohort [11], a Korean transformer-based artificial intelligence-enabled electrocardiography (AI-ECG) study [12], an Indian prospective study of lung ultrasound and NT-proBNP [13], a Korean three-hospital machine-learning study [14], a Korean non-inferiority study of an ECG-analysis application [15], a 20-centre United States NT-proBNP assay evaluation [16], an interethnic external validation in a United States critical care database [17], a prospective international comparison of two NT-proBNP assays [18], a Mayo Clinic AI-ECG diastolic function study [19], the 17-centre Prospective, Multicenter Study to Evaluate the Clinical Utility of a Novel NT‑proBNP Assay in the Emergency Department for the Diagnosis of Heart Failure (PRECISE-HF) study [20] and a Korean external validation of a regulator-cleared AI-ECG device [21].

**Table 3:** Characteristics of the 11 included studies AHF: acute heart failure; AI-ECG: artificial intelligence-enabled electrocardiography; AUC/AUROC: area under the receiver operating characteristic curve; BNP: B-type natriuretic peptide; CNN: convolutional neural network; EB: ECG Buddy; ECG: electrocardiogram; ED: emergency department; EF: ejection fraction; LV: left ventricular; LVEF: left ventricular ejection fraction; LVSD: left ventricular systolic dysfunction; MIMIC-IV: Medical Information Mart for Intensive Care IV; ML: machine learning; NPV: negative predictive value; NT-proBNP: N-terminal pro-B-type natriuretic peptide; POCUS: point-of-care ultrasound; PPV: positive predictive value.

Study [ref]	Country	Design	Population; n (target condition)	Index test	Comparator	Reference standard	Main finding
Adedinsewo et al., 2020 [11]	USA	Retrospective	ED dyspnoea; 1,606 (LVSD ≈10%)	AI-ECG CNN (LVEF ≤35%)	NT-proBNP, BNP, troponin	Echocardiography within 30 days	AI-ECG AUC 0.89 vs NT-proBNP 0.80 (p<0.0001)
Jang et al., 2024 [12]	Korea	Retrospective	ED dyspnoea; 3,105 (1,197 cardiac, 38.6%)	AI-ECG transformer network	NT-proBNP	Cardiac vs pulmonary origin at admission	AI-ECG AUC 0.938, accuracy 88.1%; superior to NT-proBNP
Sharma et al., 2025 [13]	India	Prospective	Acute breathlessness; 104 (45 AHF, 43.3%)	NT-proBNP (500 pg/mL)	Lung ultrasound; Boston criteria	Final clinical diagnosis	Sensitivity 100%, specificity 91.8%; perfect when combined with lung ultrasound
Moon et al., 2025 [14]	Korea	Retrospective	Emergency rooms; 9,119 of 19,285 screened	Machine learning (CatBoost) on ECG plus clinical data	Other ML architectures	Clinical AHF diagnosis	AUROC 0.89 internal and 0.90 external validation
Kim et al., 2025 [15]	Korea	Retrospective	ED with paired POCUS; 181 (37 LVSD, 20.4%)	AI-ECG application (EF-from-EB; LV-Dysfunction score)	NT-proBNP; POCUS	Echocardiography (LVEF <40%)	AI-ECG AUCs 0.893 and 0.884 vs NT-proBNP 0.822
Daniels et al., 2025 [16]	USA	Prospective	ED dyspnoea; 2,200 (1,095 AHF, 49.8%)	NT-proBNP (Vitros II)	None	Blinded adjudication by ≥2 clinicians	AUC 0.920; rule-out sensitivity 98.8%, NPV 97.9%
Lee et al., 2025 [17]	USA (MIMIC-IV)	Retrospective	ED visits; 22,599 (sinus rhythm subgroup 16,527)	AI-ECG image analysis (two biomarkers)	NT-proBNP	Documented LVEF <40%	AI-ECG AUCs 0.913 and 0.917 vs NT-proBNP 0.748
Belkin et al., 2025 [18]	Switzerland; international	Prospective	ED dyspnoea; 1,400 (53% AHF)	NT-proBNP–Access assay	NT-proBNP–Elecsys assay	Central adjudication	AUC 0.914 vs 0.922 (p=0.006); concordance 85.3%
Yu et al., 2025 [19]	USA	Retrospective	ED dyspnoea; 2,412 (966 cardiac, 40%)	AI-ECG diastolic function model	None	Final diagnosis on subsequent evaluation	Cardiac dyspnoea rose from 14.1% to 83.0% across grades
Allen et al., 2026 [20]	USA	Prospective	Suspected AHF; 2,384 (1,059 AHF)	Access NT-proBNP assay	Elecsys proBNP II assay	Blinded clinical events committee	AUC 0.87; rule-out sensitivity 96%, NPV 95%
Lee and Jung, 2026 [21]	Korea	Retrospective	ED; 4,529 (531 LVSD, 11.7%)	AiTiALVSD (cut-off 9.7)	None	Echocardiography (LVEF ≤40%)	AUROC 0.934; NPV 98.2%, but PPV 41.5%

Together, these studies comprised more than 40,000 emergency encounters. Five were conducted in the Republic of Korea [12,14,15,17,21], four in the United States [11,16,19,20], one in Switzerland with international recruitment [18] and one in India [13]; four were prospective [13,16,18,20] and seven retrospective [11,12,14,15,17,19,21]. Sample sizes ranged from 104 patients [13] to 22,599 ED visits from the Medical Information Mart for Intensive Care IV (MIMIC-IV) database [17]. Five studies evaluated AI-ECG alone [12,14,17,19,21], four NT-proBNP alone [13,16,18,20] and two included both tests [11,15], so four provided a direct within-cohort comparison [11,12,15,17]. Reference standards were blinded or included the central adjudication of AHF [16,18,20], a documented final clinical diagnosis [13], echocardiographic ejection fraction [11,15,17,21] or the origin of dyspnoea determined by the admitting service or subsequent evaluation [12,14,19]. Prevalence of the target condition ranged from 10% for LVSD [11] to 53% for adjudicated AHF [18].

Diagnostic Performance of NT-proBNP

Seven studies reported quantitative accuracy for NT-proBNP (Table 4) [11,13,15-18,20]. Across the three large prospective cohorts using blinded or central adjudication of AHF, discrimination was consistently high: area under the curve (AUC) 0.920 (95% confidence interval (CI): 0.909-0.931) in 2,200 patients across 20 United States EDs [16], 0.914 (0.898-0.930) and 0.922 (0.908-0.937) for the Access and Elecsys assays, respectively, in 1,400 patients with acute dyspnoea [18] and 0.87 (0.86-0.88) in 2,384 patients across 17 EDs [20].

**Table 4:** Diagnostic performance of NT-proBNP Values as published. Two rows are given where rule-out and rule-in operating points were reported separately or where two assays were compared. For Belkin et al. [18], the sensitivity shown is that of the rule-out threshold and the specificity that of the age-adjusted rule-in thresholds. AHF: Acute heart failure; AUC: area under the receiver operating characteristic curve; CI: confidence interval; COPD: chronic obstructive pulmonary disease; LVEF: left ventricular ejection fraction; LVSD: left ventricular systolic dysfunction; NPV: negative predictive value; NR: not reported; PPV: positive predictive value; sens: sensitivity; spec: specificity.

Study [ref]	Target condition	Assay and threshold	AUC (95% CI)	Sens/spec (%)	PPV/NPV (%)
Adedinsewo et al., 2020 [11]	LVSD (LVEF ≤35%)	>800 pg/mL	0.80 (0.76-0.84)	NR	NR
Sharma et al., 2025 [13]	AHF vs COPD/asthma	500 pg/mL	NR	100/91.8	89.6/100
Kim et al., 2025 [15]	LVSD (LVEF <40%)	Continuous	0.822	NR	NR
Daniels et al., 2025 [16]	Adjudicated AHF	Vitros II; rule-out <300 pg/mL	0.920 (0.909-0.931)	98.8/54.2	68.1/97.9
Daniels et al., 2025 [16]	Adjudicated AHF	Vitros II; rule-in 450/900/1,800 pg/mL	0.920 (0.909-0.931)	86.7/82.2	82.9/86.2
Lee et al., 2025 [17]	LVEF <40% (sinus rhythm)	1,796.5 pg/mL	0.748 (0.732-0.763)	79.7/59.2	NR
Belkin et al., 2025 [18]	Adjudicated AHF	Access; rule-out <300, age-adjusted rule-in	0.914 (0.898-0.930)	98.9/77.6	NR
Belkin et al., 2025 [18]	Adjudicated AHF	Elecsys; rule-out <300, age-adjusted rule-in	0.922 (0.908-0.937)	98.5/84.8	NR
Allen et al., 2026 [20]	Adjudicated AHF	Access; rule-out <300 ng/L	0.87 (0.86-0.88)	96/56	64/95
Allen et al., 2026 [20]	Adjudicated AHF	Access; rule-in 450/900/1,800 ng/L	0.87 (0.86-0.88)	85/75	73/86

The reproducible strength of NT-proBNP was rule-out. At the age-independent threshold of 300 pg/mL, sensitivity was 98.8% with a negative predictive value (NPV) of 97.9% and a negative likelihood ratio of 0.02 [16], and 96% with an NPV of 95% [20]; corresponding specificity, however, was only 54.2% and 56%. Age-stratified rule-in thresholds raised specificity to 82.2% and 75% at the cost of sensitivity falling to 86.7% and 85% [16,20]. Performance was attenuated in important subgroups: sensitivity was lower in heart failure with preserved than reduced ejection fraction (81.7% vs 92.6%) and specificity was lowest with renal insufficiency (72.5%) or prior heart failure (78.3%), although subgroup AUCs remained between 0.899 and 0.915, and roughly one in five patients fell in the grey zone, of whom 30% ultimately had AHF [16]. Assay effects were also demonstrable: Access concentrations were approximately 25% higher than Elecsys, producing minor diagnostic discordance in one in seven patients, although major mismatch was rare (0.6%) [18].

Two smaller studies reported different operating characteristics that reflect case mix rather than superior assay performance. Where the differential diagnosis was restricted to AHF versus chronic obstructive pulmonary disease or asthma, NT-proBNP at 500 pg/mL achieved 100% sensitivity and 91.8% specificity, and combination with lung ultrasound gave perfect classification [13]. Conversely, when the target condition was echocardiographic LVSD rather than AHF, NT-proBNP discrimination fell to 0.822 [15], 0.80 (0.76-0.84) [11] and 0.748 (0.732-0.763) [17]. A common decision framework nonetheless underpinned all seven studies: every one applied either the age-independent 300 pg/mL rule-out threshold, the age-stratified 450/900/1,800 pg/mL rule-in thresholds or a single explicitly stated cut-off, so that the operating point was transparent and transferable in each case.

What the AI-ECG Models Were Trained to Detect and Their Diagnostic Performance

Seven studies reported accuracy for an AI-ECG index test (Table 5) [11,12,14,15,17,19,21], with the area under the receiver operating characteristic curves (AUROCs) clustering between 0.88 and 0.94. The highest values were obtained for structural target conditions: 0.934 (0.923-0.945) for LVSD at an ejection fraction ≤40% in 4,529 ED patients [21], 0.917 (0.910-0.924) and 0.913 (0.906-0.920) for two ECG-image biomarkers predicting an ejection fraction <40% in the sinus rhythm subgroup of MIMIC-IV [17], and 0.89 (0.86-0.91) for an ejection fraction ≤35% [11]. A transformer network separating cardiac from pulmonary dyspnoea achieved an AUC of 0.938 with 88.1% accuracy, 93.0% sensitivity and 79.5% specificity [12], and a gradient-boosting model combining ECG features with clinical data achieved AUROCs of 0.89 internally and 0.90 externally [14].

**Table 5:** Diagnostic performance of AI-ECG, with the model target label and operating threshold used by each study Values as published; studies evaluating more than one biomarker are shown on separate rows. Four distinct target labels were used across the seven studies, and only three of the seven reported an explicit numerical operating threshold, in contrast to the standardised NT-proBNP decision limits shown in Table 4. Yu et al. [19] reported the discriminative gradient as the frequency of cardiac dyspnoea across AI-ECG diastolic grades (14.1%, 20.8%, 62.2% and 83.0%). AHF: Acute heart failure; AI-ECG: artificial intelligence-enabled electrocardiography; AUROC: area under the receiver operating characteristic curve; CI: confidence interval; EB: ECG Buddy; ECG: electrocardiogram; EF: ejection fraction; LV: left ventricular; LVEF: left ventricular ejection fraction; LVSD: left ventricular systolic dysfunction; NPV: negative predictive value; NR: not reported; PPV: positive predictive value; sens: sensitivity; spec: specificity.

Study [ref]	Architecture and input	Target label used to train or define the model	Model output and operating threshold	AUROC (95% CI)	Sens/spec (%)	PPV/NPV (%)
Adedinsewo et al., 2020 [11]	Convolutional neural network; 12-lead waveform	Echocardiographic LVEF ≤35%	Probability of LVSD; threshold not numerically reported	0.89 (0.86-0.91)	73.8/87.3	NR
Jang et al., 2024 [12]	Transformer network; 12-lead waveform	Cardiac vs pulmonary origin of dyspnoea at admission	Probability of cardiac origin; threshold not numerically reported	0.938	93.0/79.5	89.0/86.4
Moon et al., 2025 [14]	CatBoost gradient boosting; ECG features plus clinical variables	Clinical diagnosis of acute heart failure	Probability of AHF; threshold not numerically reported	0.89 internal; 0.90 external	NR	NR
Kim et al., 2025 [15]	Commercial application; ECG image (EF-from-EB)	Echocardiographic LVEF <40%	Predicted ejection fraction (continuous)	0.893	NR	NR
Kim et al., 2025 [15]	Commercial application; ECG image (LV-Dysfunction score)	Echocardiographic LVEF <40%	Continuous score	0.884	NR	NR
Lee et al., 2025 [17]	Commercial application; ECG image (LV Dysfunction score)	Documented LVEF <40%	Continuous score; threshold 0.352	0.913 (0.906-0.920)	83.1/85.5	NR
Lee et al., 2025 [17]	Commercial application; ECG image (LVEF-ECG)	Documented LVEF <40%	Predicted ejection fraction; threshold 49.138	0.917 (0.910-0.924)	85.5/83.8	NR
Yu et al., 2025 [19]	Diastolic function model; 12-lead waveform	Echocardiographic diastolic function and filling pressure	Ordinal grade 0-3	NR	NR	NR
Lee and Jung, 2026 [21]	AiTiALVSD residual network; 12-lead waveform	Echocardiographic LVEF ≤40%	Continuous score 0-100; prespecified cut-off 9.7	0.934 (0.923-0.945)	88.3/83.5	41.5/98.2

Critically, these algorithms were not detecting a shared electrocardiographic finding, and the criteria they applied were not the same across studies. None of the seven models used human-defined ECG criteria at all; each was trained end-to-end to reproduce a reference label, and four different labels were used. Five models were trained on echocardiographic systolic function, itself defined at two different ejection fraction thresholds, ≤35% [11] or <40% and ≤40% [15,17,21]; one was trained on the admitting service's classification of dyspnoea as cardiac or pulmonary [12]; one on a clinical diagnosis of AHF supported by ejection fraction and natriuretic peptide data [14]; and one on echocardiographic diastolic function and filling pressure [19]. Model inputs also differed, with three algorithms analysing the digitised waveform and three an image of the printed tracing. The practical consequence is that the seven AUROCs in Table 5 are not estimates of a single quantity, and that a positive AI-ECG result carries a different meaning in each study.

The reporting of operating thresholds differed sharply from that of NT-proBNP. Whereas every natriuretic peptide study applied a transparent and transferable decision limit, only three of the seven AI-ECG studies reported an explicit numerical threshold: a prespecified cut-off of 9.7 on a 0-100 scale [21], and thresholds of 0.352 for a continuous dysfunction score and 49.138 for a predicted ejection fraction [17]. The remaining four reported discrimination without stating the operating point at which their sensitivity and specificity were obtained [11,12,14,19], which prevents a reader from applying the model at the bedside and is reflected in the CLAIM appraisal below.

Where thresholds were stated, operating characteristics were those of a screening rather than a confirmatory test. At a cut-off of 9.7 and an LVSD prevalence of 11.7%, sensitivity was 88.3% and NPV 98.2%, but positive predictive value of only 41.5%, so that a negative result made LVSD improbable, whereas a positive result required echocardiographic confirmation [21]. Discrimination in that cohort was preserved in haemodynamically unstable patients, with AUROCs of 0.895 (0.854-0.937) at a shock index ≥0.9 and 0.885 (0.786-0.984) in the 75 hypotensive patients, the latter designated exploratory [21].

One study departed from the LVSD paradigm by applying an AI-ECG model of diastolic function and filling pressure to 2,412 ED patients with dyspnoea, of whom 966 (40%) had a cardiac cause. Model-assigned grades were normal in 922 (38.2%), grade 1 in 245 cases (10.2%), grade 2 in 1,192 (49.4%) and grade 3 in 53 (2.2%), and the proportion with cardiac dyspnoea rose monotonically across these grades from 14.1% to 20.8%, 62.2% and 83.0% [19]. This gradient is notable because elevated filling pressure, rather than reduced ejection fraction, is the physiological hallmark of AHF and the substrate that natriuretic peptide release also reflects.

Direct Within-Cohort Comparisons

Four studies applied both index tests to the same patients and are therefore the only source of direct comparative evidence (Table 6) [11,12,15,17]. AI-ECG discriminated better in every instance, and significantly so in the three reporting a paired statistical comparison: AUC 0.89 (0.86-0.91) versus 0.80 (0.76-0.84) at a threshold of 800 pg/mL (p<0.0001) [11]; 0.893 and 0.884 for the ECG-derived ejection fraction and LV-Dysfunction score versus 0.822 (p=0.017 and p=0.030), with the ECG-derived ejection fraction additionally non-inferior to point-of-care ultrasound at a 0.05 AUC margin [15]; and 0.913 and 0.917 versus 0.748 in the sinus rhythm subgroup of 22,599 ED visits, a difference of approximately 0.17 AUC units (all p<0.001) preserved in the overall population [17]. A transformer-based model likewise outperformed NT-proBNP for identifying cardiac dyspnoea, although neither the NT-proBNP AUC nor a paired statistic was reported [12]. In all four, the reference standard was echocardiographic LVSD or the admitting-service classification of dyspnoea; no included study compared the two tests against a centrally adjudicated AHF reference standard. All other contrasts that might be drawn between Tables 4 and 5 are indirect, involve different patients, prevalences and reference standards, and are reported here as description rather than as comparative evidence.

**Table 6:** Direct within-cohort comparisons of AI-ECG and NT-proBNP Only these four studies applied both index tests to the same patients against the same reference standard and are therefore the only source of direct comparative evidence in this review; all other contrasts between Tables 4 and 5 are indirect. In every direct comparison, the target condition was left ventricular systolic dysfunction or the cardiac origin of dyspnoea rather than centrally adjudicated acute heart failure. AI-ECG: artificial intelligence-enabled electrocardiography; AUC: area under the receiver operating characteristic curve; ED: emergency department; LVEF: left ventricular ejection fraction; LVSD: left ventricular systolic dysfunction; MIMIC-IV: Medical Information Mart for Intensive Care IV; NT-proBNP: N-terminal pro-B-type natriuretic peptide.

Study [ref]	Cohort	Target condition	AI-ECG AUC	NT-proBNP AUC	Paired comparison
Adedinsewo et al., 2020 [11]	Mayo Clinic ED; 1,606	LVSD (LVEF ≤35%)	0.89 (0.86-0.91)	0.80 (0.76-0.84)	AI-ECG superior, p<0.0001
Jang et al., 2024 [12]	Korean tertiary ED; 3,105	Cardiac vs pulmonary dyspnoea	0.938	Not reported numerically	AI-ECG reported as superior; no paired statistic given
Kim et al., 2025 [15]	Korean ED; 181	LVSD (LVEF <40%)	0.893; 0.884	0.822	AI-ECG superior, p=0.017 and 0.030
Lee et al., 2025 [17]	MIMIC-IV ED; 22,599	LVEF <40% (sinus rhythm)	0.913; 0.917	0.748 (0.732-0.763)	AI-ECG superior, all p<0.001

Risk of Bias

The QUADAS-2 assessment, visualised with the robvis tool [22], is shown in Figure 2. Eight studies were judged at overall low risk of bias [11-13,15,16,18-20] and three at high risk [14,17,21]. All three high-risk judgements were driven by the patient-selection domain, in which enrolment was conditioned on the availability of the reference standard, and two were compounded by concerns in the flow-and-timing domain arising from intervals of up to 30 days between the index ECG and the reference echocardiogram [17,21]. Some concerns were additionally raised for the index test, reference standard and flow domains in the machine-learning study whose target condition was defined by routinely coded clinical diagnosis [14], and for the reference standard in the database-derived external validation [17]. The index-test domain was rated low in 10 of the 11 studies, reflecting the fact that most algorithms and their operating thresholds had been fixed before the validation cohort was assembled.

**Figure 2:**
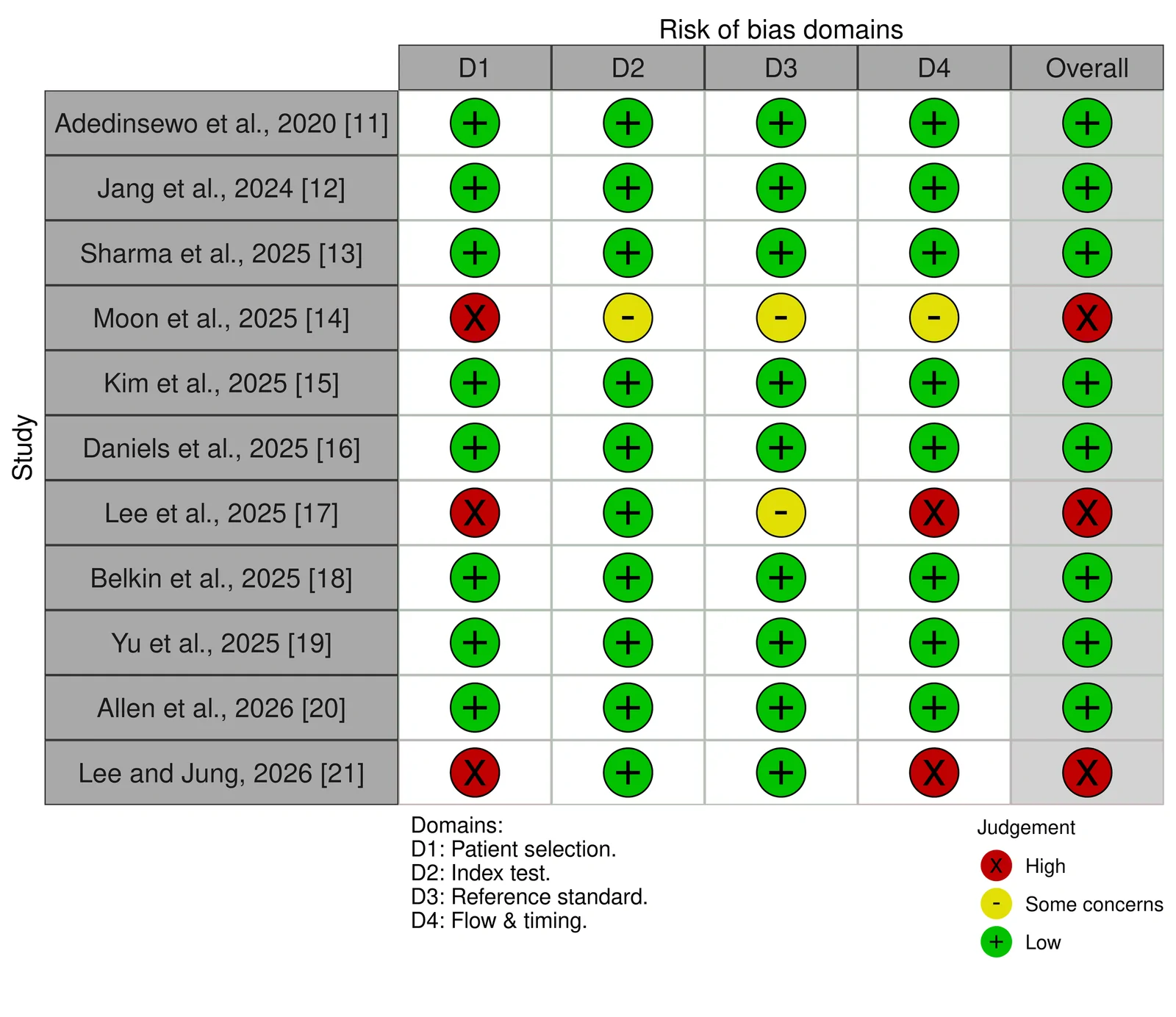
Risk of bias of the 11 included studies Assessed with the QUADAS-2 tool and visualised with robvis [22]. D1: patient selection; D2: index test; D3: reference standard; D4: flow and timing.

Reporting Completeness of the Artificial Intelligence Studies

Reporting completeness of the seven studies evaluating a machine-learning index test was appraised against the CLAIM 2024 checklist (Figure 3) [10]. Reporting was uniformly complete for the title and abstract and for the introduction, and was largely complete for evaluation metrics and validation strategy, which were fully reported in five of seven studies. Weaknesses were clustered under three domains. Model description and version were only partially reported in five studies [11,12,15,17,19], most often because the algorithm was a proprietary or commercially licensed application described by reference to a prior publication rather than specified in the report itself [11,15,17,19]. Data sources, ground-truth definition and data partitioning were partially reported in four studies [12,14,17,19]. Transparency, encompassing data and code availability alongside declaration of developer involvement, was fully reported in only one study [21]. Training and hyperparameter tuning were not applicable in the five studies that applied a previously fixed algorithm without retraining [11,15,17,19,21]. Overall, three studies were judged to have fully reported the majority of applicable domains [11,15,21] and four to have reported them partially [12,14,17,19]. These reporting gaps align closely with the missing operating thresholds documented in Table 5.

**Figure 3:**
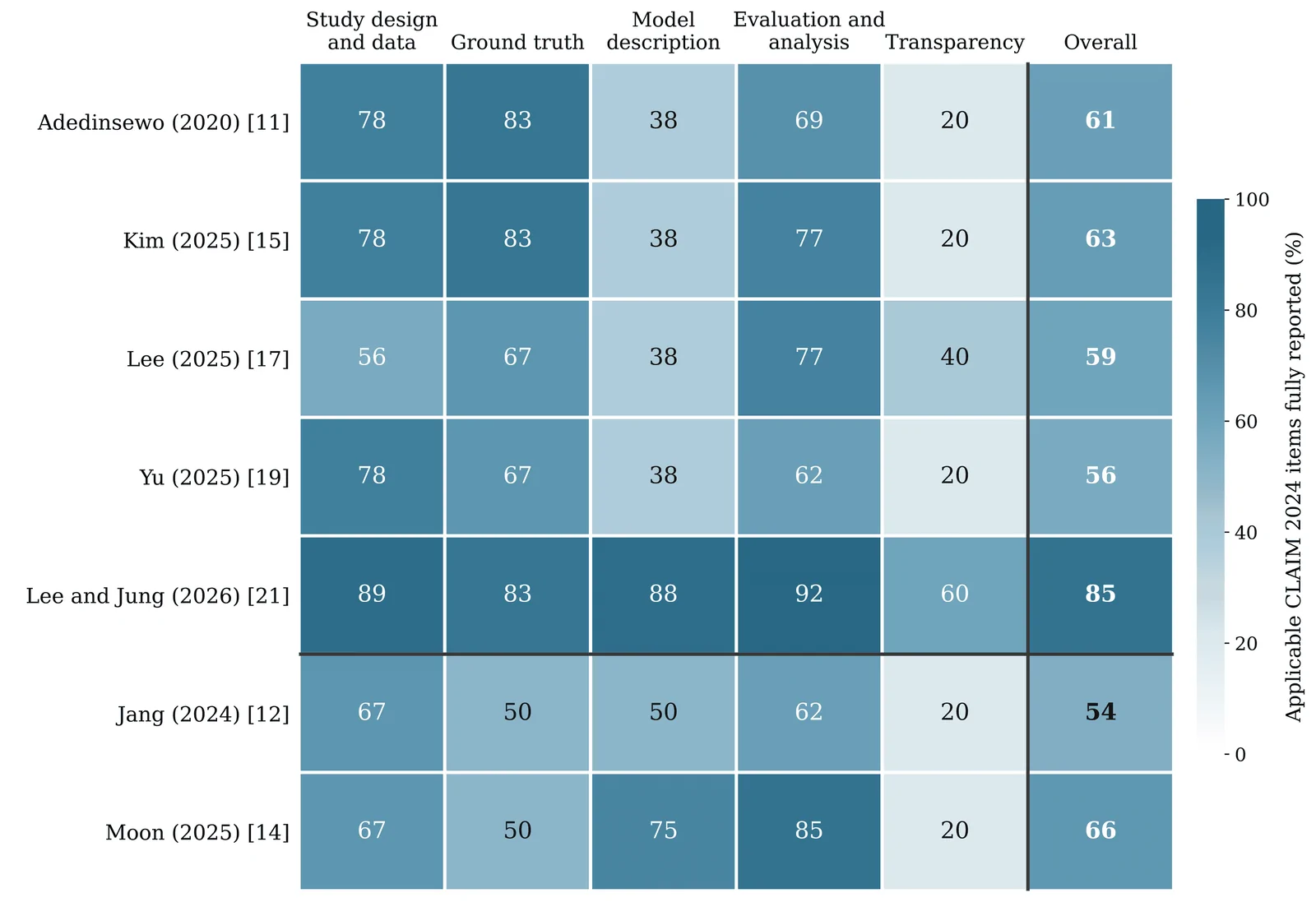
Reporting completeness of the seven artificial intelligence studies CLAIM: Checklist for Artificial Intelligence in Medical Imaging [10]. Domains: C1 title and abstract; C2 introduction; C3 study design, setting and eligibility; C4 data sources, ground truth and partitions; C5 model description and version; C6 training and hyperparameter tuning; C7 evaluation metrics and validation; C8 transparency, limitations and availability.

Discussion

Principal Findings

This systematic review of 11 primary studies comprising more than 40,000 emergency encounters found that NT-proBNP and AI-ECG both perform well in the ED, but that they do so for different target conditions and with different operating characteristics. NT-proBNP demonstrated highly reproducible discrimination for centrally adjudicated AHF, with AUCs between 0.87 and 0.92 across three independent multicentre prospective cohorts totalling nearly 6,000 patients [16,18,20], and an essentially unmatched capacity to exclude the diagnosis, with sensitivities of 96%-99% and negative predictive values of 95%-98% at the 300 pg/mL threshold. AI-ECG achieved numerically higher AUROCs, between 0.88 and 0.94, and outperformed NT-proBNP in all four direct within-cohort comparisons [11,12,15,17]. Those comparisons were, however, made against echocardiographic LVSD or an admitting-service proxy for cardiac dyspnoea rather than against adjudicated AHF. The superiority of AI-ECG is therefore real within the studies that report it, though not, on the present evidence, directly transferable to the clinical question of whether a breathless patient in front of the emergency physician has AHF.

NT-proBNP: Consistency and Its Limits

The consistency of the NT-proBNP results is one of the more striking findings of this synthesis. Discrimination was almost identical whether measured with the Vitros NT-proBNP II assay across 20 United States EDs [16], with the Access assay across 17 EDs [20], or with the Access and Elecsys assays in an international European cohort [18], and the range of AUCs (0.87-0.92) sits comfortably within the interval reported two decades earlier in the foundational PRIDE and ICON-RELOADED studies [4,5]. Reproducibility across eras, continents, case mixes and analytical platforms is powerful evidence of biological rather than statistical validity. It is equally important, however, that specificity at the rule-out threshold was only 54%-56%, that approximately one in five patients occupied the grey zone, and that sensitivity fell to 81.7% in heart failure with preserved ejection fraction [16]. Recognition of these limitations has driven decision-support approaches such as the Collaboration for the Diagnosis and Evaluation of Heart Failure (CoDE-HF) tool, which combines NT-proBNP as a continuous measure with routine clinical variables and, in an individual patient data meta-analysis of 10,369 patients, delivered better calibrated probability estimates than any fixed threshold, identifying 40.3% of patients without prior heart failure as low probability with a negative predictive value of 98.6% [23]. The most productive direction of travel for natriuretic peptide testing is therefore not a better assay but a better model of how to interpret the assay in an individual patient.

AI-ECG: Accuracy for a Different Target Condition

The AI-ECG literature is at an earlier stage of maturity. A prior systematic review of AI-ECG screening for LVSD across all care settings identified 15 studies with a median AUC of 0.90 and a median sensitivity of 83.3%, values almost exactly reproduced by the ED-specific studies in the present review [24]. That convergence indicates that AI-ECG detects LVSD with reliable, transportable accuracy. The difficulty is that LVSD is not AHF: roughly half of patients presenting with AHF have a preserved ejection fraction and would be classified as normal by an LVSD-detection algorithm, whereas many patients with chronic LVSD present with dyspnoea from pneumonia or exacerbated airways disease and would be flagged as positive. This is a target-condition mismatch rather than a performance deficiency, and it is the single most important interpretive caveat in this literature. Its practical consequence is visible in the operating characteristics: at an LVSD prevalence of 11.7%, a well-validated algorithm delivered an NPV of 98.2% but a PPV of only 41.5% [21].

The heterogeneity of model targets documented in Table 5 compounds the problem. Because the seven algorithms were trained on four different labels and evaluated at thresholds that three of them did not disclose, a positive AI-ECG result does not carry a common meaning across studies, and the field currently lacks anything comparable to the standardised 300, 450, 900 and 1,800 pg/mL decision limits that make NT-proBNP portable between institutions. Establishing consensus operating points for a defined target condition is a prerequisite for clinical adoption, not an afterthought.

Towards Algorithms That Target the Syndrome

Two included studies point towards how the mismatch might be resolved. Rather than estimating ejection fraction, the Mayo Clinic group applied an AI-ECG model of diastolic function and filling pressure and showed a monotonic rise in the proportion with cardiac dyspnoea across model-assigned grades, from 14.1% to 83.0% [19]. Because elevated left ventricular filling pressure is the shared physiological substrate of both symptomatic congestion and natriuretic peptide release, an algorithm that estimates filling pressure is conceptually far closer to a true AHF test than one that estimates ejection fraction. Similarly, a gradient-boosting model that combined ECG features with routinely available clinical variables achieved an external-validation AUROC of 0.90 for AHF itself rather than for a structural surrogate [14], suggesting that modest clinical context added to the ECG signal may close much of the gap between substrate detection and syndrome recognition. The earliest ECG-based deep learning work in heart failure was in fact framed around identification of the syndrome rather than the ejection fraction [25], and returning to that framing with contemporary architectures and adjudicated labels is a logical next step.

Why the Two Tests Are Complementary

NT-proBNP is a state marker, released from cardiomyocytes in proportion to instantaneous wall stress and therefore rising and falling with the haemodynamic decompensation that defines AHF. AI-ECG, as currently implemented, is predominantly a trait marker, detecting the electrical signature of chronic structural remodelling that constitutes the substrate on which decompensation occurs. A patient with severe chronic LVSD presenting with pulmonary embolism will have a strongly positive AI-ECG and may have only a modestly elevated NT-proBNP; a patient with hypertensive flash pulmonary oedema and a structurally near-normal heart may have a strikingly elevated NT-proBNP and an unremarkable AI-ECG. Neither test is wrong, because they answer different questions. This framing predicts that the combination should outperform either alone, a hypothesis no included study formally tested and the most obvious near-term research opportunity. Proof of principle exists in the adjacent literature, where combining NT-proBNP with lung ultrasound produced perfect classification of AHF versus airways disease in one included cohort [13].

Direct Versus Indirect Comparisons and Statistical Caveats

The strength of inference in this review differs sharply between two classes of comparison, and conflating them would be the most likely source of overinterpretation. Only the four studies in Table 6 applied both index tests to the same patients, verified against the same reference standard, with a paired analysis in three of them; only these support a claim about relative accuracy, and even then the claim holds for LVSD or cardiac dyspnoea rather than for AHF. Every other contrast between Tables 4 and 5 is indirect. Indirect comparison of diagnostic accuracy is hazardous for reasons that are well understood but easily overlooked: the AUC is not prevalence-independent in practice, because it is estimated in a specific case mix, and cohorts in which the target condition occurred in 10% of patients [11] are not exchangeable with cohorts in which it occurred in 53% [18]; spectrum effects mean that accuracy measured in a cohort selected for having undergone echocardiography will exceed that obtainable at unselected triage; and an AUC computed against echocardiographic ejection fraction and one computed against adjudicated AHF are estimates of different parameters that happen to share a scale.

For these reasons, no pooled estimate, hierarchical summary receiver operating characteristic curve or between-study significance test was generated, and the numerical ranges quoted throughout the Results section should be read as the span of observed values rather than as a summary effect with a confidence interval. A formal diagnostic test accuracy meta-analysis would have been feasible arithmetically but not interpretable clinically, since the resulting summary sensitivity and specificity would describe a target condition that no single included study actually evaluated.

A further consideration cuts across both index tests and, unusually, the two biases run in opposite directions. In all three high-quality prospective natriuretic peptide cohorts, adjudicators had access to standard-of-care natriuretic peptide results when assigning the reference diagnosis [16,18,20]; this incorporation of the index test into the reference standard would be expected to inflate the apparent accuracy of NT-proBNP. Conversely, the direct comparisons used reference standards that structurally favour AI-ECG, because echocardiographic ejection fraction is precisely what those algorithms were trained to reproduce. The honest conclusion is that the true relative accuracy of the two tests for adjudicated AHF is not yet known.

Clinical Implications

The practical implications for emergency practice favour early adoption of AI-ECG in a defined role. An ECG is already recorded at triage in most patients with dyspnoea, requires no venepuncture, incurs essentially zero marginal cost once an algorithm is deployed and returns a result within seconds rather than the hour or more typical of laboratory natriuretic peptide turnaround. That AI-ECG discrimination is preserved in patients with a shock index ≥0.9 and in hypotensive patients [21] matters precisely because these are the patients in whom decisions about fluid administration, inotropic support and disposition must be made before laboratory results return. That an ECG-image application analysed on a smartphone was non-inferior to physician-performed point-of-care ultrasound for detecting LV dysfunction [15] suggests a genuine role where sonographic expertise, echocardiographic equipment or natriuretic peptide assays are unavailable. None of this displaces natriuretic peptide testing, which the current European and North American guidelines and the most recent focused update all continue to recommend for every patient with suspected AHF [1,26,27]. Positioned as a rapid first-pass triage tool that prioritises patients for echocardiography and expedites cardiology involvement, AI-ECG has a defensible and immediately implementable role.

Implementation, Reporting and Generalisability

Several implementation issues temper enthusiasm, and the CLAIM appraisal quantifies one of them. Although study design and evaluation were generally well reported, model specification was only partially reported in five of seven AI studies and transparency regarding data and code availability in six of seven. When the algorithm is a proprietary application described only by reference to an earlier publication, independent replication is impossible and post-deployment drift cannot be audited by the users who depend on it. Generalisability is also only partially established: most algorithms evaluated here were developed in Korean or United States tertiary cohorts, and although interethnic external validation in a US database preserved discrimination [17], performance in African, South Asian and Latin American ED populations is unknown. The ED acquisition environment is hostile to signal quality, with movement, pain, tachypnoea and variable electrode placement all degrading the input; that discrimination was maintained despite these conditions is reassuring but should not be assumed universal [21]. Calibration, as distinct from discrimination, was rarely reported, yet it is calibration that determines whether a model's probability output can be acted upon at the bedside.

Knowledge Gaps and Research Agenda

This review identifies several specific and addressable gaps. First, no study has compared AI-ECG with NT-proBNP against a blinded, centrally adjudicated AHF reference standard in a prospective ED cohort; until that is done, the claim that AI-ECG outperforms NT-proBNP for the diagnosis of AHF is not evidentially supported, however consistently it is reported for LVSD. Second, no study evaluated the incremental value of adding AI-ECG to NT-proBNP, or of embedding both within a probabilistic framework analogous to CoDE-HF [23]. Third, AI-ECG performance in heart failure with preserved ejection fraction - the phenotype in which NT-proBNP is weakest and clinical need greatest - remains almost unexamined in the ED; the diastolic-function models that would address it have so far been reported only descriptively [19], and the most rigorous recent external validation of an artificial intelligence model for this phenotype was based on echocardiography rather than the ECG [28]. Fourth, no randomised or interventional study has assessed whether AI-ECG-guided pathways alter time to diagnosis, time to diuresis, length of stay, rehospitalisation or mortality; all available evidence is diagnostic accuracy evidence, which is necessary but insufficient for implementation. Fifth, health-economic evaluation is absent despite the fact that the principal argument for AI-ECG is one of cost and access rather than accuracy. Sixth, as the CLAIM appraisal demonstrates, consistent adoption of contemporary reporting standards [10], including explicit statement of the target label and the operating threshold, would materially improve the interpretability of this literature. Addressing these gaps requires prospective, multicentre, blinded diagnostic studies in unselected ED dyspnoea populations, followed by pragmatic randomised evaluation of complete diagnostic pathways rather than isolated index tests.

Limitations

This review has several limitations. The main limitation was the heterogeneity of the included studies in target condition, index-test threshold, and reference standard, which prevented meta-analysis, pooled estimates, hierarchical summary receiver operating characteristic analysis, and statistical comparison between studies; consequently, publication bias could not be formally assessed by funnel plot, and small-study effects favouring positive AI-ECG results cannot be excluded. As set out above, only four studies provided direct within-cohort comparison, and all remaining contrasts are indirect and descriptive. The review was not prospectively registered in PROSPERO or an equivalent register, although eligibility criteria were specified in a written protocol before searching and are reported in Table 1, and the full search strategies are reported in Table 2. Eligibility was restricted to English-language, peer-reviewed publications from 2020 onward, with the foundational PRIDE and ICON-RELOADED studies discussed only as background and preprints excluded. Although eight of 11 studies had low risk of bias on QUADAS-2, the three high-risk studies involved AI-ECG evaluations conditioned on echocardiography availability, potentially inflating performance. CLAIM assessment was limited to domain-level evaluation based on published reports and should therefore be considered a screening-level assessment rather than a definitive audit. Several studies also involved authors, funding or employment from assay or algorithm manufacturers, introducing potential optimism bias. Finally, five of 11 cohorts and four algorithms originated in the Republic of Korea, while four cohorts came from two US academic systems, limiting the geographical and ethnic diversity and generalizability of the evidence base.

## Conclusions

In adults presenting to the emergency department with suspected AHF, NT-proBNP remains the more rigorously validated diagnostic test, with consistent discrimination across multicentre prospective cohorts and strong rule-out ability at 300 pg/mL, although its specificity is modest and performance is reduced in preserved ejection fraction and obesity, with a substantial grey zone. AI-ECG showed higher discrimination than NT-proBNP in all four direct within-cohort comparisons and offers practical advantages in speed, cost, availability and independence from phlebotomy, including in haemodynamically unstable patients; however, its evidence is mainly for LVSD rather than AHF itself, the seven algorithms were trained on four different target labels, and only three reported an explicit operating threshold. Thus, the two tests should currently be considered complementary rather than interchangeable: NT-proBNP reflects the acute haemodynamic state, whereas AI-ECG reflects the underlying structural substrate. NT-proBNP should therefore remain the primary rule-out test for AHF, while AI-ECG may serve as a rapid triage tool to prioritise echocardiography and specialist assessment, particularly where resources are limited. Prospective blinded head-to-head studies using adjudicated AHF, standardisation of target labels and operating thresholds, evaluation of combined biomarker-AI pathways, and outcome-based trials are needed before AI-ECG can replace natriuretic peptide testing.
